# Extremely low frequency-pulsed electromagnetic fields affect proangiogenic-related gene expression in retinal pigment epithelial cells

**DOI:** 10.22038/ijbms.2018.25023.6214

**Published:** 2019-02

**Authors:** Morteza Oladnabi, Abouzar Bagheri, Mozhgan Rezaei Kanavi, Abbas Azadmehr, Anvarsadat Kianmehr

**Affiliations:** 1 Stem Cell Research Center, Golestan University of Medical Sciences, Gorgan, Iran; 2 Ischemic Disorders Research Center, Golestan University of Medical Sciences, Gorgan, Iran; 3 Department of Clinical Biochemistry and Genetics, Molecular and Cell Biology Research Center, Faculty of Medicine, Mazandaran University of Medical Sciences, Sari, Iran; 4 Tissue Engineering Research Center, Shahid Beheshti University of Medical Sciences, Tehran, Iran; 5 Immunology Department, Babol University of Medical Sciences, Babol, Iran; 6 Medical Cellular and Molecular Research Center, Golestan University of Medical Sciences, Gorgan, Iran; 7 Department of Medical Biotechnology, School of Advanced Technologies in Medicine, Golestan University of Medical Sciences, Gorgan, Iran

**Keywords:** ELF-PEMF, Gene expression, Proangiogenic factors, Quantitative real-time PCR, RPE cells

## Abstract

**Objective(s):**

It is known that extremely low frequency-pulsed electromagnetic fields (ELF-PEMF) influence multiple cellular and molecular processes. Retinal pigment epithelial (RPE) cells have a significant part in the emergence and pathophysiology of several ocular disorders, such as neovascularization. This study assessed the impact of ELF-PEMF on the proangiogenic features of RPE cells.

**Materials and Methods:**

Primary cultured RPE cells were treated with ELF-PEMF (50 Hz) for three days. Using ELISA assay, we evaluated the effects of treatment on RPE cell proliferation and apoptosis. Also, RT-PCR was used to determine the gene expression of proangiogenic factors, such as matrix metalloproteinase-2 (MMP-2), MMP-9, vascular endothelial growth factors receptor 2 (VEGFR-2), hypoxia-inducible factor 1 (HIF-1α), VEGFA, cathepsin D, connective tissue growth factor (CTGF), E2F3, tissue inhibitors of metalloproteinases 1 (TIMP-1), and TIMP-2.

**Results:**

No noticeable changes were observed in cell proliferation and cell death of ELF-PEMF-exposed RPE cells, while transcript levels of proangiogenic genes (HIF-1α, VEGFA, VEGFR-2, CTGF, cathepsin D, TIMP-1, E2F3, MMP-2, and MMP-9) increased significantly.

**Conclusion:**

RPE cells are important for homeostasis of the retina. ELF-PEMF increased the gene expression of proangiogenic factors in RPE cells, which highlights concerns about the impact of this treatment on human health.

## Introduction

Extremely low frequency-pulsed electromagnetic fields (ELF-PEMF) are composed of a broad spectrum of oscillating electromagnetic fields with a defined frequency of less than 100 Hz. ELF-PEMFs are deemed critical for public health due to the widespread application of electrical power at 50 or 60 Hz in most communities. Indeed, constant exposure to ELF-PEMFs, mostly with unknown effects, raises concerns about their potential hazards.

Ongoing studies indicate that ELF-PEMFs can affect several cellular and molecular processes (1-3). Angiogenesis is an extremely complex process, which is controlled physiologically by the microenvironment and genetically by changes in several oncogenes or tumor suppressor genes, representing a critical process in normal states of growth, development, wound healing, and essential steps of tumor progression and metastatization (4, 5).

Retinal pigment epithelial (RPE) cells create a simple cuboidal layer of cells, located behind the photoreceptor (PR) cells, serving as a supporting context for PR cells, which results in visual perception. Basic and clinical studies revealed that a primary disorder in RPE cells can lead to visual cell death and blindness (6). In neovascularization development, RPE cells play an essential role by producing several factors, such as vascular endothelial growth factors (VEGFs) (7, 8), VEGF receptors (VEGFRs) (9), connective tissue growth factor (CTGF), tissue inhibitors of metalloproteinases (TIMPs), matrix metalloproteinases (MMPs) (10), and cathepsin D (11), which contribute to paracrine signaling between RPE and capillaries (12). 

RPE cells constitutively produce VEGF, which is the most powerful angiogenic promoter, and is involved in the pathogenesis of multiple disorders related to ocular neovascularization, including diabetic retinopathy (DR) and age-related macular degeneration (AMD) (13-15). VEGF/VEGFR signaling has consequences, including secretion or activation of matrix-degrading proteinases (MMPs and cathepsins), CTGF, and E2Fs (16-18). CTGF has an integral function in regulating the extracellular matrix turnover, and a relationship is established between CTGF and choroidal neovascularization (CNV) (13, 19). Matrix-degrading proteinases and their inhibitors (TIMPs) are implicated in physiological (growth and development, morphogenesis, tissue remodeling, angiogenesis, and fibrosis) and pathological processes (cancer, DR, and AMD) (10, 20).

It is important to know whether ELF-PEMFs affect gene expression, as it will clarify the impact of electrical equipment on molecular mechanisms of biological processes. The present study aimed to clarify if 50-Hz ELF-PEMF exposure affects gene expression of proangiogenic factors or proliferation of RPE cells.

## Materials and Methods

***Culture and sample preparation***

The study protocols were approved by the Ophthalmic Research Center Ethics Committee, Shahid Beheshti University of Medical Sciences (Tehran, Iran). An informed consent was obtained from the participants, and the Central Eye Bank of Iran approved the experimental use of tissues from the donated eye. 

**Figure 1:**
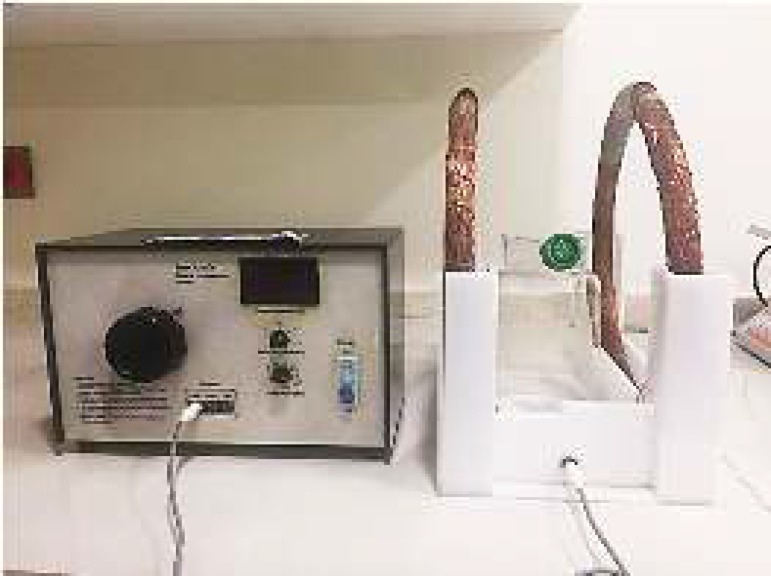
The magnetic field generator (Amen Ara ® Ni200A) composed of Helmholtz coils and current controller system. The Helmholtz coils consists of two identical coils with diameter of 22 cm with 22 cm axial separation distance. By altering the current delivered to coils, the magnetic field generated by coils was adjusted to desired value (1 mT). The magnetic field intensity was calibrated at 1 mT by a field strength meter

After isolating RPE cells from healthy globes of human neonates, they were cultured in a 1:1 DMEM:F12 medium (Sigma, Germany), containing 10% fetal bovine serum. After reaching 60% confluence in the cultures, the cells in the treatment group were exposed to a pulsed electromagnetic field (1 mT, 50 Hz) for 8 hr a day for three days. The magnetic field generator (Amen Ara® Ni200A) is indicated in Figure 1, which is composed of Helmholtz coils and a current controller system. The Helmholtz coils consisted of two identical coils with a diameter of 22 cm and 22 cm axial separation distance. By altering the current delivered to coils, the coil-generated magnetic field was adjusted to the desired value (1 mT). The pulse interval was 20 mSec (frequency, 50 Hz), with a pulse duration of 1 mSec. The magnetic field was 1 mT at the axis of two coils where the samples were located. The magnetic field intensity was calibrated at 1 mT by a field strength meter (21).

***ELF-PEMF treatment***

The ELF-PEMF exposure system was constructed as a solenoid electromagnet (ring-shaped; diameter, 22 cm; Helmholtz coil), consisting of 800 wire loops. All the experiments were carried out at a frequency of 50 pulses per second in a pulsed magnetic field at 1 mT for three days (8 hr per day). The pulse interval was 20 mSec (frequency, 50 Hz), with a pulse duration of 1 mSec.

***Immunocytochemistry***

After culturing RPE cells in 24-well plates, they were fixed for 10 min in −10°C methanol. TritonX-100 (0.25%) was used for permeabilizing the cells. Then, 1% bovine serum albumin in phosphate-buffered saline (PBS) was used to block the cells for 60 min at room temperature. To confirm the epithelial cell /RPE identity, staining was performed using a rabbit RPE65 polyclonal antibody to label RPE microsomal membranes, along with a specific mouse anti-human cytokeratin 8/18 monoclonal antibody labeling epithelial cells (1:1000; Santa Cruz, CA). 

For the detection of culture immunoreactivity to primary antibodies, fluorescein isothiocyanate (FITC)-conjugated antibodies were used, including goat anti-mouse and anti-rabbit IgGs (1:400; Santa Cruz). Then, the slides were incubated in 1.5 mg/ml of DAPI (Santa Cruz) for 10 min to stain the nuclear DNA. To evaluate the slides, we used a fluorescence microscope (Olympus IX71, Japan) with 460-nm and 520-nm filters for antibodies conjugated with DAPI and FITC, respectively.

***ELISA assay for cell proliferation***

The RPE cells were incubated in 200 μl of complete medium in 96-well plates (10000 cells/well) at 37 ^°^C (5% CO_2_) until reaching nearly 60% confluence. To determine if ELF-PEMF changes cell proliferation, bromodeoxyuridine was added following ELF-PEMF exposure, and the proliferation assay was carried out, as outlined by the manufacturer (Roche, Germany).

***Cell death ELISA assay***

After culturing RPE cells in 96-well plates containing 200 μl of complete medium (10000 cells/well), incubation was performed at 37°C (5% CO_2_) until reaching nearly 60% confluence. By exposing RPE cells to the cell death assay kit, cytotoxicity was evaluated following ELF-PEMF exposure, as described by the manufacturer (Roche, Germany).

***RNA isolation***

For extracting total RNA from treated and control cells, TRIzol (Ambion, USA) was used. The samples reacted for 5 min at room temperature. For extracting RNA, chloroform was used, and to precipitate RNA, 500 µl of isopropanol was added and rinsed with 75% Ethanol. Following that, RNA was dissolved in nuclease-free water. Spectrophotometric analysis was carried out to determine the purity and concentration of RNA. To confirm RNA integrity, agarose gel electrophoresis was carried out, preceding ethidium bromide staining.

***RT–PCR assay***

A reverse transcriptase kit (Qiagen, Germany) was used for the reverse transcription reaction. EvaGreen (Solis BioDyne, Estonia) was also used to perform quantitative RT–PCR. The assay included denaturation for 15 min at 95 ^°^C (one cycle); denaturation, amplification, and quantification for 10 sec at 95 ^°^C, for 25 sec at 58–64 ^°^C, and for 20 sec at 72 ^°^C (40 cycles); followed by a melting curve (a gradual increase from 72 ^°^C to 95 ^°^C). Table 1 presents the primer sequences for RT-PCR assay.

***Data analysis***

The results of cell death and proliferation assays are expressed as mean±SD of three analyses. The RT–PCR assay was carried out in duplicate as three independent experiments. To determine intergroup differences, student’s t-test was used. The significance level was 0.05.

## Results

***RPE cell isolation and characterization***

**Figure 2:**
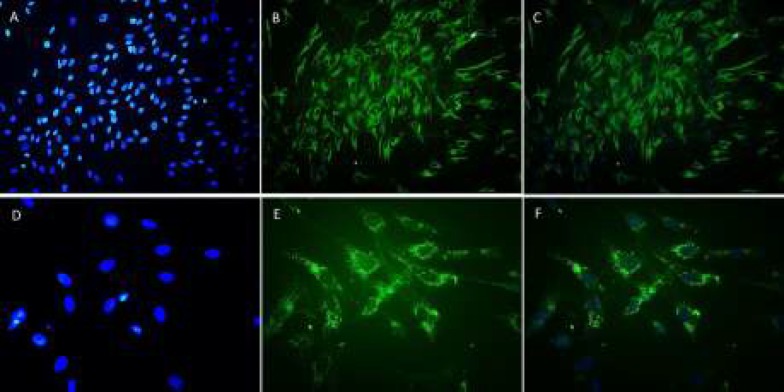
Immunocytochemistry of retinal pigment epithelial (RPE) cells indicating RPE cell identity and culture purity. To confirm the epithelial origin of the cultures, cytokeratin 8/18 expression was assessed, and to confirm that isolated cells were RPE cells, RPE65 was surveyed. A: Nuclei stained blue with 4,6-diamidino-2-phenyindole dihydrochloride (DAPI). B: RPE cells stained positively for the fluoresceinisothiocyanate (FITC)-conjugated cytokeratin antibody (green). C: Merged image (FITC-labeled cytokeratin and DAPI; 100X). D: DAPI-stained RPE cell nuclei (blue). E: RPE cells stained positively for the RPE65 antibody (green). F: Merged image (FITC-labeled RPE65 and DAPI; 400X)

After reaching 80% confluence in primary RPE cells culture, the culture medium was removed. Then, methanol was used to fix the slides, which were then subjected to the immunocytochemistry (ICC) protocol (22). The identity of RPE cells was confirmed, as they expressed cytokeratin 8/18 and RPE65 (Figure 2).

***Effects of ELF-PEMF on RPE proliferation***

**Figure 3:**
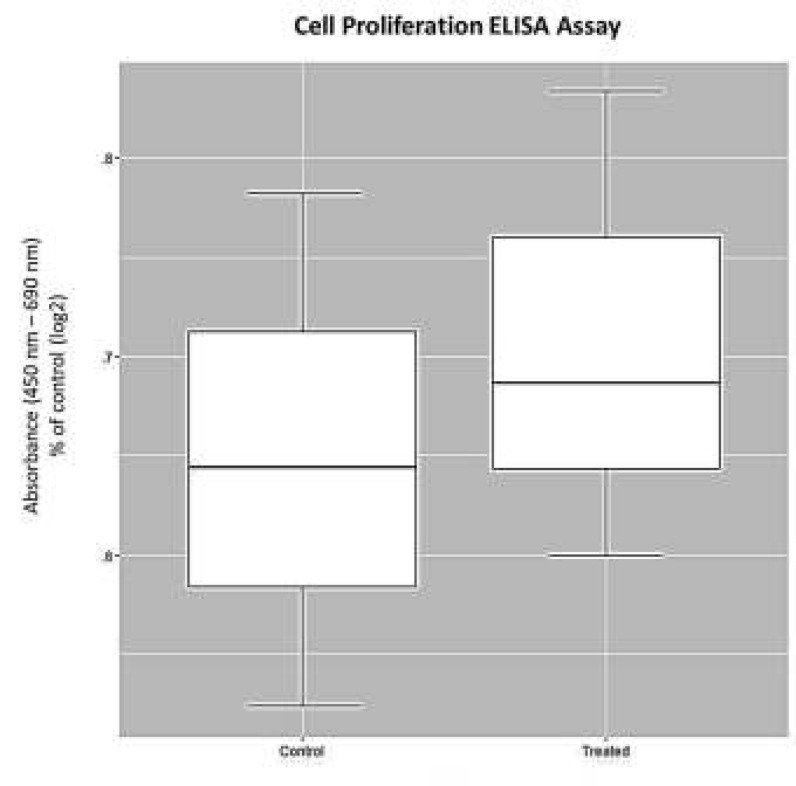
Effect of extremely low frequency-pulsed electromagnetic fields (ELF-PEMF) on the proliferation rate of human retinal pigment epithelial (RPE) cells. RPE cells exposed to ELF-PEMF. After 3 days of exposure (8 hr per day), and the cells without exposure as the control, the cultures were harvested and proliferation assay was performed according to the manufacturer’s instructions. Proliferation rate of RPE cells did not change under exposure to ELF-PEMF (*P >* 0.05)

The control and ELF-PEMF-treated cells were evaluated by ELISA assay regarding cell proliferation. ELF-PEMF did not change RPE proliferation in comparison with the control cells (Figure 3).

***Cytotoxic effects on RPE cells***

**Figure 4:**
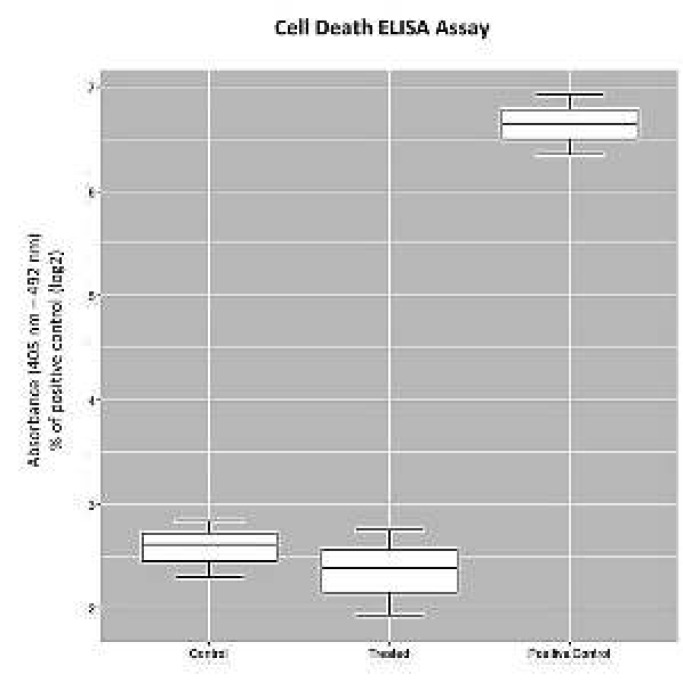
Determination of the cytotoxic effects of extremely low frequency-pulsed electromagnetic fields (ELF-PEMF) on human retinal pigment epithelial (RPE) cells. RPE cells exposed to ELF-PEMF for 3 days (8 hr per day) and the cells without exposure were considered as the control. After exposing to ELF-PEMF, cultures were harvested and subjected to cell death assay according to the manufacturer’s instructions. Results indicated that ELF-PEMF did not impose cytotoxic effects on treated cultures when compared to the positive control (*P<*0.05)

The potential cytotoxic effect of ELF-PEMF was evaluated using ELISA assay. ELF-PEMF did not show any cytotoxic impact on RPE cells in comparison with positive controls using the cell death detection kit (Figure 4).

**Figure 5:**
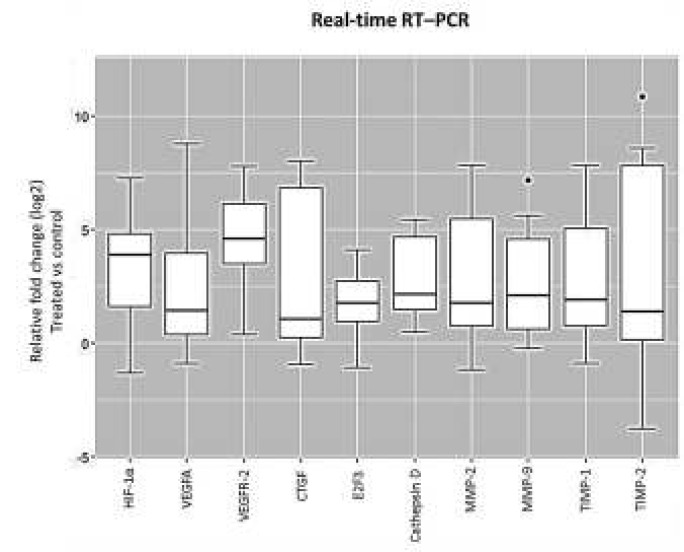
Box plot analysis of hypoxia-inducible factor 1 (HIF-1α), vascular endothelial growth factors A (VEGFA), vascular endothelial growth factors receptor 2 (VEGFR-2), and connective tissue growth factor (CTGF), E2F3, cathepsin D, matrix metalloproteinase-2 (MMP-2), MMP-9, tissue inhibitors of metalloproteinases 1 (TIMP-1) and TIMP-2 expression in retinal pigment epithelial (RPE) cell cultures exposed to extremely low frequency-pulsed electromagnetic fields (ELF-PEMF). Cultures exposed to ELF-PEMF and the cells without exposure were considered as treatment and the control, respectively. After 3 days, RNA was extracted, and gene expression analysis was performed with quantitative real-time RT–PCR as described in the methods section. mRNA levels were normalized to Glyceraldehyde 3-phosphate dehydrogenase (GAPDH) and presented as log2 fold change of the control values. ELF-PEMF increased gene expression of HIF-1α, VEGFA, VEGFR-2, CTGF, E2F3, MMP-2, MMP-9 and TIMP-1 in treated cell cultures compared to the control (*P<*0.05). However, gene expression of cathepsin D and TIMP-2 were not altered (*P>*0.05)

**Table1:** Primer sequences used in real-time RT-PCR analysis

Name	Forward	Reverse
GAPDH	ACAGTCAGCCGCATCTTC	CTCCGACCTTCACCTTCC
VEGFA	GGAGGGCAGAATCATCACGAA	GGTCTCGATTGGATGGCAGT
CTGF	TGAAGCTGACCTGGAAGAGA	GCTCAAACTTGATAGGCTTGG
MMP-2	TGGCAAGTACGGCTTCTGTC	TTCTTGTCGCGGTCGTAGTC
MMP-9	TGGAGGTTCGACGTGAAG	AGTTGCAGGATGTCATAGGTC
TIMP-1	TGCGGATACTTCCACAGGTC	GCATTCCTCACAGCCAACAG
TIMP-2	AAGAGCCTGAACCACAGGTA	GAGCCGTCACTTCTCTTGAT
E2F3	GAAAGCCCCTCCAGAAACAAG	GCTATGTCCTGAGTTGGTTGAAG
HIF-1α	AACTGGAGACACAATCATATCTTTAG	TTCAGCGGTGGGTAATGGAG
Cathepsin D	TCTCTGTCCTACCTGAATGTCAC	AATGTCGGGAGGAACGTGTC
	VEGFR-2 Qiagen, Germany, QT00073640	

VEGFR-2 primers were pre-designed commercially supplied from Qiagen.

Matrix metalloproteinase (MMP), Vascular endothelial growth factors receptor (VEGFR), Hypoxia-inducible factor 1 (HIF-1α), Vascular endothelial growth factors A (VEGFA), Connective tissue growth factor (CTGF), Tissue inhibitors of metalloproteinases (TIMP), Glyceraldehyde-3-phosphate dehydrogenase (GAPDH) .

***RT–PCR assay***

RT–PCR assay was performed to determine if ELF-PEMF changed the expression level of HIF-1α, cathepsin D, MMP-2, MMP-9, CTGF, VEGFA, VEGFR-2, E2F3, TIMP-1, and TIMP-2 genes in RPE cells. After normalizing mRNA expression to glyceraldehyde-3-phosphate dehydrogenase (GAPDH) mRNA level, differences in expression were determined using the established standard curve and efficiency (E) for the primer sets. ELF-PEMF exposure resulted in the increased expression of HIF-1α, VEGFA, VEGFR-2, CTGF, cathepsin D, E2F3, TIMP-1, MMP-2, and MMP-9, while TIMP-2 expression did not show any changes (Figure 5).

## Discussion

In our study, three days of ELF-PEMF exposure (1 mT, 50 Hz, 8 hr on/16 hr off) to RPE cells did not have any cytotoxic or proliferative effects on RPE cells. However, significant upregulation of proangiogenic factors, HIF-1α, VEGFA, VEGFR-2, CTGF, cathepsin D, E2F3, TIMP-1, MMP-2, and MMP-9, was reported.

So far, this is the first study regarding ELF-PEMF effects on RPE cells. Electromagnetic fields with various parameters (e.g., magnetic flux density, frequency, and exposure time) may have different consequences. For instance, Maziarz *et al*. showed that stimulation of stem cells by EMF might have positive effects, such as regeneration, homeostasis, and wound healing, or negative effects, such as degeneration and carcinogenesis (23). Consequently, ELF-PEMF has positive effects on proangiogenic molecules, which can be a reason for increasing abnormal ocular angiogenic diseases including diabetic retinopathy and AMD.

Diseases including DR, retinopathy of prematurity, and ischemic retinal-vein occlusion involve in intraocular neovascularization. Also, neovascularization is the cause of one of two forms of macular degeneration related to AMD. Visual loss occurs in neovascularization through leakage of blood and serum of fragile new vessels. Studies show that diseases like AMD and DR have prominent global burdens. AMD has an increasing rate in the world, and according to Wong *et al*., 8.7% of the world population had AMD in 2014, which is speculated to increase to 196 million in 2020 and 288 million in 2040. Therefore, considering public concerns regarding the possible adverse effects of growing consumer devices and power lines, the impact of ELF-EMF on human health is of great importance (24, 25).

A key molecule in many ocular diseases such as DR and oxidative AMD is VEGFA (26, 27). HIF-1α increases gene expression of VEGFA by activating its promoter, and VEGFA promotes cell proliferation, migration, and survival by signaling through its related receptor, VEGFR-2 (28). VEGFA upregulates CTGF, which is a profibrotic and proangiogenic factor in different organs and is linked to angiogenesis and pathological fibrosis, such as vitreoretinal disorders (DR and AMD) (19, 29). 

The expression of many downstream mediators is triggered by VEGFA and CTGF, including proteases (e.g., cathepsins and MMPs) and their inhibitors (e.g., TIMPs). Major pathological mechanisms in processes of ocular diseases (e.g., proliferative retinopathy and neovascularization) include the RPE-interphotoreceptor matrix and Bruch’s membrane disintegration. Alterations in matrix-degrading proteinase expression in the mentioned diseases suggest that these enzymes may cause pathogenesis of ocular disorders. In early phases of angiogenesis, degradation of extracellular matrix proteins is a common phenomenon by MMPs and cathepsin proteases (13).

RPE cells significantly contribute to the homeostasis of the retina (30). Human primary RPE cell cultures provide substantial prospects as an *in vitro* model for analyzing the influence of different agents at cellular and molecular levels. HIF-1α was significantly increased in ELF-PEMF-exposed RPE cells. Also, VEGFA, specially VEGFR-2 and CTGF increased, which was followed by augmentation in gene expression of cathepsin D, E2F3, TIMP-1, MMP-2, and MMP-9. All the mentioned molecules are linked to active neovascularization (10, 26, 31). They are also effective factors in triggering or progression of neovascularization.

## Conclusion

In our study, treatment of RPE cells continued for 24 hr over three days, which is shorter than actual exposure to environmental ELF-PEMFs and maybe insufficient for determining the cytotoxic or proliferative effects of ELF-PEMF. However, more inclusive studies are necessary to specify the possible effects and underlying mechanisms of ELF-EMF on life quality. 
